# Three-dimensional assessment of vascular cooling effects on hepatic microwave ablation in a standardized ex vivo model

**DOI:** 10.1038/s41598-022-21437-4

**Published:** 2022-10-12

**Authors:** C. A. Neizert, H. N. C. Do, M. Zibell, C. Rieder, D. Sinden, S. M. Niehues, J. L. Vahldiek, K. S. Lehmann, F. G. M. Poch

**Affiliations:** 1 Department of General and Visceral Surgery-Campus Benjamin Franklin, Charité-Universitätsmedizin Berlin, Corporate Member of Freie Universität Berlin, Humboldt-Universität zu Berlin, and Berlin Institute of Health, Berlin, Germany; 2 Fraunhofer Institute for Digital Medicine MEVIS, Bremen, Germany; 3 Department of Radiology-Campus Benjamin Franklin, Charité-Universitätsmedizin Berlin, Corporate Member of Freie Universität Berlin, Humboldt-Universität zu Berlin, and Berlin Institute of Health, Berlin, Germany

**Keywords:** Surgical oncology, Hepatocellular carcinoma

## Abstract

The aim of this study was a three-dimensional analysis of vascular cooling effects on microwave ablation (MWA) in an ex vivo porcine model. A glass tube, placed in parallel to the microwave antenna at distances of 2.5, 5.0 and 10.0 mm (A–V distance), simulated a natural liver vessel. Seven flow rates (0, 1, 2, 5, 10, 100, 500 ml/min) were evaluated. Ablations were segmented into 2 mm slices for a 3D-reconstruction. A qualitative and quantitative analysis was performed. 126 experiments were carried out. Cooling effects occurred in all test series with flow rates ≥ 2 ml/min in the ablation periphery. These cooling effects had no impact on the total ablation volume (p > 0.05) but led to changes in ablation shape at A–V distances of 5.0 mm and 10.0 mm. Contrary, at a A–V distance of 2.5 mm only flow rates of ≥ 10 ml/min led to relevant cooling effects in the ablation centre. These cooling effects influenced the ablation shape, whereas the total ablation volume was reduced only at a maximal flow rate of 500 ml/min (p = 0.002). Relevant cooling effects exist in MWA. They mainly depend on the distance of the vessel to the ablation centre.

## Introduction

Microwave ablation (MWA) is a minimally invasive in situ procedure that is used as an alternative to surgical resection for treating primary and secondary liver malignancies^1–4^. In MWA, microwaves induce an electromagnetic field at a frequency of 915 or 2450 MHz^1,5^. At these frequencies oscillating water molecules within the target tissue lead to a temperature increase up to 120 °C. Emerging changes in tissue composition like carbonization raise electrical impedance. This limits the effective application of other minimally invasive techniques such as radiofrequency ablation (RFA), which rely on resistive heating^6^. In contrast, the electromagnetic field of MWA can propagate through desiccated or charred tissue so that higher temperatures and larger, more uniform ablation volumes are achieved^3,7^.

As it delivers energy through dipole moments effects induced by the propagation of the electromagnetic waves, MWA is also considered to be less susceptible to the “heat sink effect” of naturally occurring liver vessels compared to RFA, in which thermal diffusion plays a more significant role^8–10^. The heat sink effect may influence ablation shape and volume, increasing the risk of incomplete ablation and local tumour recurrence. Accurate knowledge of these cooling effects with reference to contributing factors is relevant in clinical routine to ensure oncological safety. Two different types of cooling effects are known: the diffuse and the directional cooling effect^11,12^. The diffuse cooling effect results from the general perfusion of the liver. Especially smaller blood vessels lead to a diffuse dissipation of heat energy, mainly influencing the total ablation volume but not the ablation shape^12^. In contrast, the directional cooling effect is caused by larger hepatic vessels passing through or immediately around an ablation. These vessels extract energy from the ablation at specific points, influencing both ablation shape and volume^11,12^. Incomplete ablation is possible in these cases, resulting in local tumour recurrence in clinical routine. For this reason, it is essential to record both ablation shape and volume when evaluating MWA.

Nevertheless, studies examining the heat sink effect in MWA are rare. Ringe et al. identified both the distance of the MWA antenna to the vessel and the vascular flow rate as risk factors for an incomplete ablation^13^. However, most ex and in vivo studies are insufficient in displaying the effects of vascular cooling realistically. Without medical imaging techniques such contrast-enhanced computed tomography (CECT) or magnetic resonance imaging (MRI) study findings are mostly based on two-dimensional (2D) data analyses. Ablations from standard probes are naturally three-dimensional (3D) and ellipsoidal in shape^14^. Algorithms on measurements are necessary to analyse the full extent of the heat sink effect on ablation shape and volume.

### Objective

The objective of this study was a three-dimensional analysis of cooling effects in hepatic MWA in a standardized ex vivo porcine model. The influence of antenna to vessel distance as well as vascular flow rate on ablation shape and volume were evaluated.

## Results

A total of 148 microwave ablations were carried out at 100 W for 5 min at room temperature. However, 22 ablations had to be repeated due to naturally occurring and interrupting large liver vessels (n = 10), technical errors (automatic termination of the ablation, n = 3) or exceeding of the ablation beyond the liver sample (n = 9). Each ablation was subdivided into 2 mm slices, so that 1498 ablation slices were examined in total.

The energy loss caused by the cooling vessel was determined by measuring the change in temperature of the cooling liquid in all test series with flow rates ≥ 100 ml/min before and after MWA. The initial temperature of the cooling liquid prior to an ablation was 21.8 °C (18.8–23.0 °C). A median temperature increase of 0.6 K was recorded after MWA. 4.9–9.1% of the applied energy was absorbed by the cooling vessel when the flow rate was 100 ml/min. This energy loss increased to 13.9–19.2% in all test series with a flow rate of 500 ml/min. This energy dissipation was most pronounced when the cooling vessel was located immediately adjacent to the antenna (A–V distance: 2.5 mm). The impact of these energy losses on ablation shape and volume is described in the subsequent qualitative and quantitative analysis.

### Qualitative analysis

**Figure 1:**
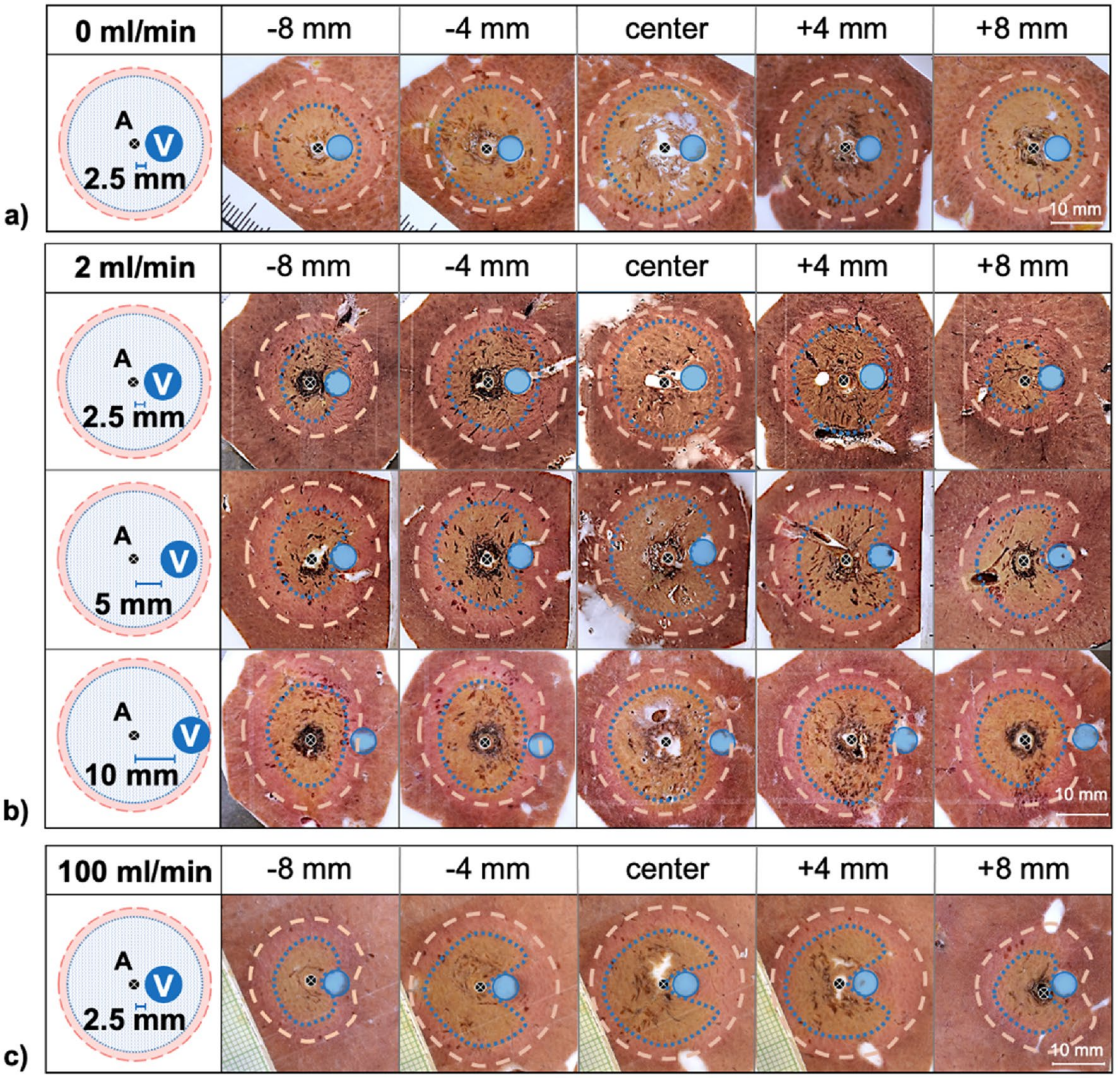
Example cross sections of ablations with a perfusion rate of 0 ml/min (**a**), 2 ml/min (**b**) and 100 ml/min (**c**). The ablation centre as well as cross sections of the ablation periphery (± 4 mm and ± 8 mm) are shown. The typical ablation zones [inner WZ (blue dotted), outer RZ (white dashed)] could be observed in all test series. No change in ablation shape of the WZ could be observed in the ablation centre at an A–V distance of 2.5 mm at a flow rate of 2 ml/min. Relevant cooling effects occurred only at a higher flow rate of 100 ml/min at an A–V distance of 2.5 ml/min (**c**). All other cross sections showed indentations of the clinically relevant WZ around the cooling vessels (V) at higher A–V distances of 5.0 and 10.0 mm independent of the flow rate.

The shapes of all ablation slices were evaluated macroscopically and compared to a round and homogenous ablation with no vessel perfusion (0 ml/min) (Fig. 1a). A WZ (white zone) and a RZ (red zone) could be identified macroscopically in all ablations. At a close A–V distance of 2.5 mm, no cooling effects were observed in the ablation centre up to flow rates of < 100 ml/min (Fig. 1b). In contrast, flow rates ≥ 1 ml/min already showed cooling effects in form of an indentation at A–V distances of 5.0 mm and 10.0 mm in the ablation centre. Distinct cooling effects were visible in the outer third of the ablation in all test series. Based on the qualitative evaluation, the distance between the ablation centre and the respective cooling vessel, rather than the distance between the antenna and the cooling vessel seems to be relevant for the occurrence of cooling effects.

### Quantitative analysis

The total ablation volumes for the A–V distances at each flow rate are shown in Table 1. Only a close A–V distance of 2.5 mm and a maximum flow rate of 500 ml/min led to a volume reduction of the ablation (p = 0.002). For all other measurements, neither the A–V distance nor the flow rate had an impact on the ablation volume. This finding is contrary to the qualitative analysis where cooling effects had a relevant influence on ablation shape in all test series with perfusion. Consequently, a sole quantitative assessment of the ablation volume alone is not sufficient to depict cooling effects. Therefore, a reverse analysis of the cooling volume, which was derived by subtracting the actual ablation volume from an idealized ablation with no cooling effects, was performed (Table 1). Cooling effects occurred from ≥ 10 ml/min onward for an A–V distance of 2.5 mm. Flow rates ≥ 2 ml/min led to cooling volumes at A–V distances of 5.0 and 10.0 mm. An indirect evaluation of the cooling volume seems to be more sensitive than a direct assessment of the ablation volume. However, the cooling volume still does not allow any statement about the exact spatial extent of the cooling effect.

**Table 1:** Ablation volume and cooling volume (ml) depending on the vascular flow rate. The cooling volume was derived by subtracting the achieved ablation volume from an idealized ablation defined by the respective maximum radii of each ablation slice. The test series with perfusion were compared to the test series without perfusion (median (min/max); *p ≤ 0.008; ^(^*^)^p = 0.008–0.05).

	0 ml/min	1 ml/min	2 ml/min	5 ml/min	10 ml/min	100 ml/min	500 ml/min
**A–V distance: 2.5 mm**							
Ablation volume	7.2 (6.2/8.6)	7.4 (6.2/8.4)	5.6^(^*^)^ (4.7/7.0)	6.2 (5.5/7.4)	6.3 (5.3/6.5)	5.8^(^*^)^ (5.1/6.6)	5.4* (5.2/6.1)
Cooling volume	0.6 (0.4/1.0)	0.8 (0.0/0.9)	1.0 (0.7/1.4)	1.0^(^*^)^ (0.7/1.3)	1.2* (0.9/1.8)	1.6* (1.4/1.8)	1.8* (1.4/1.9)
Cooling portion	8%	11%	18%	16%	19%	28%	33%
**A–V distance: 5.0 mm**							
Ablation volume	6.7 (5.0/9.0)	6.2 (4.4/8.8)	6.9^(^*^)^ (5.5/7.2)	4.8 (4.3/6.1)	4.6 (2.2/6.2)	4.4 (3.9/7.0)	5.2 (3.9/6.1)
Cooling volume	0.6 (0.3/1.1)	1.0 (0.8/1.2)	1.4* (0.9/1.9)	1.4* (1.1/1.7)	1.4^(^*^)^ (0.8/1.9)	1.7* (1.7/2.6)	1.7* (1.0/1.9)
Cooling portion	9%	16%	20%	29%	30%	39%	33%
**A–V distance: 10 mm**							
Ablation volume	6.7 (4.7/8.7)	7.1 (5.3/7.7)	6.9 (5.4/7.7)	6.1 (4.3/7.6)	5.9 (4.0/7.6)	6.1 (5.3/7.4)	5.6 (3.7/5.9)
Cooling volume	0.8 (0.5/1.1)	1.0 (0.7/1.2)	1.6* (0.9/1.8)	1.1 (0.7/1.3)	1.3* (0.8/1.7)	1.3^(^*^)^ (0.8/1.7)	1.1^(^*^)^ (0.9/1.4)
Cooling portion	12%	14%	23%	18%	22%	21%	20%

### Semi-quantitative analysis

**Figure 2:**
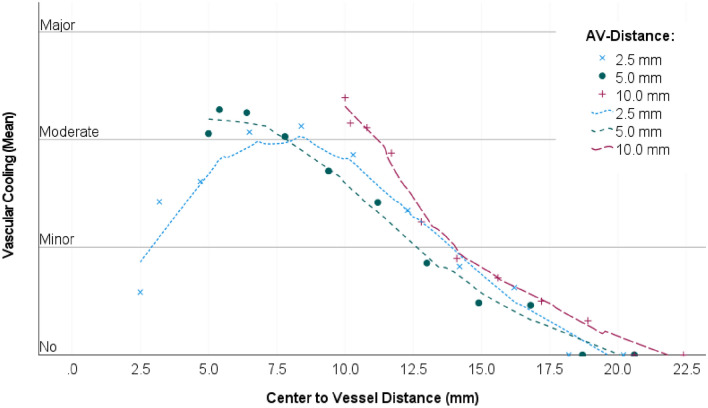
Semiquantitative analysis of vascular cooling effects dependent on the distance between the vessel to the ablation centre (all flow rates combined). The mean values for the cooling effects of all 1498 ablation slices were assessed. A local regression (LOESS) was used to plot the regression curves (dotted lines). A dependency between the distance of the vessel to the ablation centre was identified. Moderate cooling effects were seen when the vessel was located close to the ablation border (≈ 7.5 mm from the ablation centre). Minor cooling effects were seen in the ablation centre.

**Figure 3:**
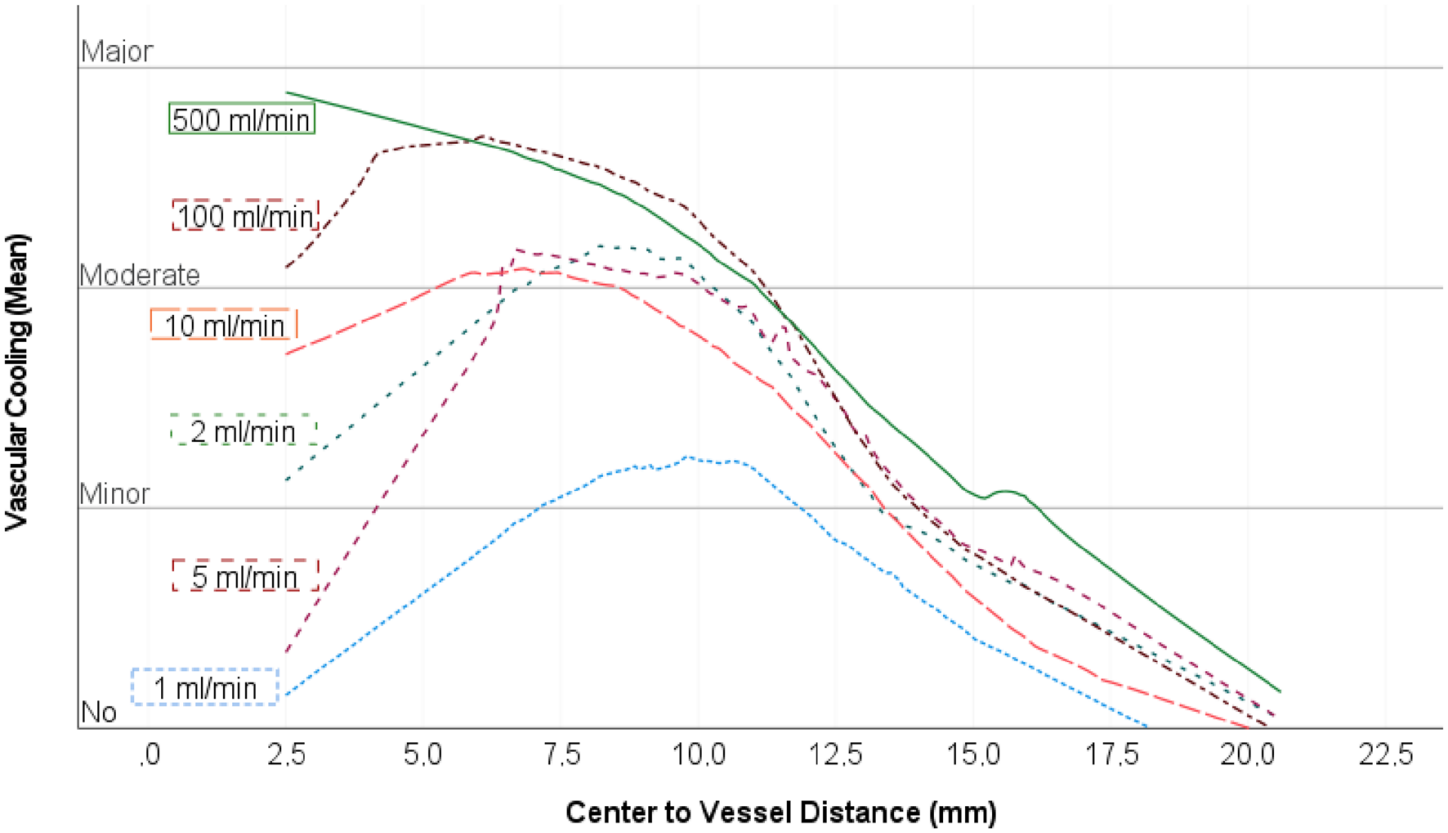
Local regression curves (LOESS; comparable to Fig. 4) depending on the flow rates (1–500 ml/min). The averaged individual values, which are the basis of the regression analysis, have been omitted for visual clarity. No or minor cooling effects could be demonstrated for the test series with a perfusion rate of 1 ml/min. From a flow rate of 2 ml/min, moderate cooling effects occurred at a distance of 7.5 mm from the ablation centre in all test series. Moderate to severe cooling effects occurred at the ablation centre above a flow rate of 10 ml/min.

A semi-quantitative analysis, subdividing cooling effects into four different types (Fig. 5), was performed to evaluate the spatial extent of the cooling effect. Figure 2 shows the cooling effect (as classified in Fig. 5) dependent on the distance between the vessel to the ablation centre for each of the A–V distances. Little or no cooling effects occurred in the ablation centre when the antenna was placed close to the vessel (A–V distance of 2.5 mm). However, moderate cooling effects were identified at all A–V distances as the vessel approached the ablation border (≈ 7.5 mm from the ablation centre point). This implies that cooling vessels in the vicinity of the ablation border had the strongest impact on the ablation shape. Figure 3 shows the semi-quantitative analysis of cooling effects as a function of flow velocity. Moderate cooling effects occurred from flow rates ≥ 10 ml/min in the ablation centre. Relevant cooling effects occurred regardless of flow velocity (≥ 2 ml/min) when the vessel was placed close to the ablation border (≈ 7.5 mm from the ablation centre point). The cooling effect decreased again with increasing distance of the vessel to the ablation border. Therefore, results of the semi-quantitative analysis were consistent with the pre-described qualitative findings.

**Figure 4:**
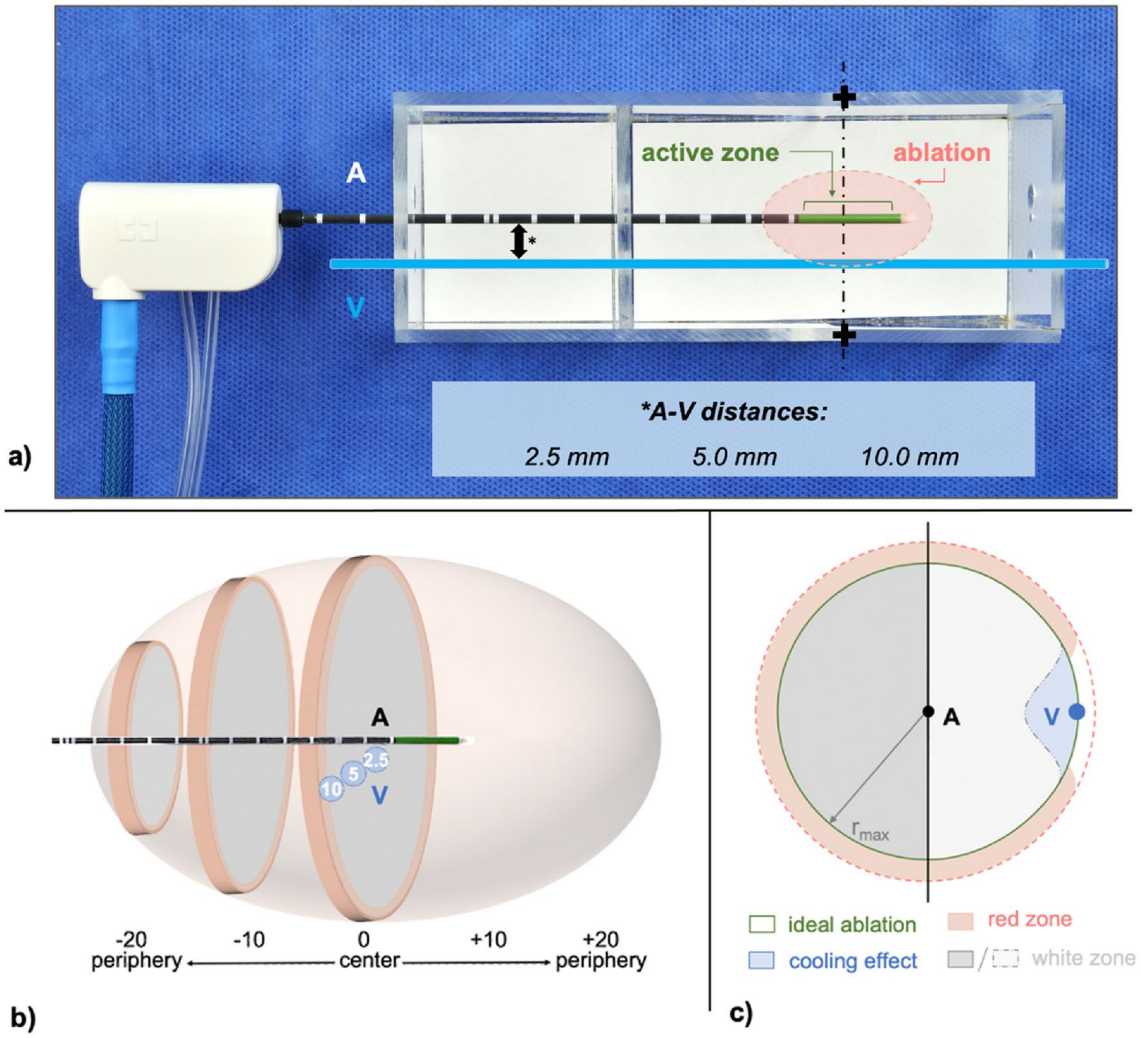
(**a**) Experimental setup using a custom-made aiming device allowing exact positioning of the microwave antenna (A) and glass tube, which simulated a natural vessel (V). Three different antenna to vessel (A–V) distances (*) were evaluated. All ablations were initially cut at the largest cross-sectional area (+) which is expected at the centre of the active zone of the microwave antenna. (**b**) Ablations were then bisected every 2 mm from the ablation centre (0). Each ablation half could be divided into a maximum of ten slices (− 20/+ 20). (**c**) Only the ablation half containing the vessel (V) was used for a quantitative analysis. Cooling effects were determined by subtracting the actual ablation area from an idealized ablation, which was calculated with the maximum ablation radius (r_max_).

## Discussion

In this study, the influence of the heat sink effect on MWA shape and volume was assessed three-dimensionally in a standardized hepatic ex vivo porcine model. We could show that vascular cooling effects exist even in up to date, third generation microwave systems. These cooling effects were largely independent from the perfusion rate, as they already occurred at minimal vascular flow rates ≥ 2 ml/min. Cooling effects were visible especially at the ablation border resulting in a flattening of the ablation. However, this peripheral flattening did not affect the total ablation volume. In contrast, cooling effects were less likely to appear centrally at low flow volumes (< 10 ml/min) when the antenna was placed immediately adjacent to a vessel (A–V distance of 2.5 mm). Nevertheless, these central cooling effects influenced the ablation shape and led to a reduction of the total ablation volume at increased flow rates ≥ 500 ml/min. In summary, cooling effects were dependent on the distance between the ablation centre and the vessel. Cooling effects in the ablation centre were less likely.

In recent years, hepatic MWA has become an established minimally invasive therapy in the treatment of colorectal liver metastases and small to medium sized HCC^1,2,6,15^. Vessels in close vicinity to the target tissue are a known risk factor for incomplete tumour ablation and subsequent tumour recurrence of thermal ablation techniques such as MWA and RFA^6,16,17^. Even though MWA is considered to be less influenced by the heat sink effect due to faster active tissue heating and higher ablation temperatures in comparison to RFA, increased local recurrence rates have been described in cases of perivascular tumour location^18,19^. The analysis of distinct cooling effects on patients is complex. Standardized ex vivo studies are needed to investigate this effect in more detail. Most studies have examined the cooling effect two-dimensionally through a sole analysis of the ablation centre^7,13,20^. Antenna to vessel distance, vascular flow rate and vessel diameter were identified as risk factors for incomplete ablation^11,13,21^. In addition to these previous 2D studies, we investigated the cooling effect three-dimensionally ex vivo using a glass tube as a vascular substitute^22^. Consistent with previous studies, we identified the antenna-vessel spacing as an important factor that considerably affects the cooling effect. However, we were able to show that the antenna to vessel distance only has an indirect influence on the cooling effect. Instead, the distance of the vessel to the ablation centre (geometric ablation midpoint) seems to be the decisive factor. When the vessel was located in the ablation periphery, even minimal flow rates ≥ 2 ml/min resulted in considerable indentations of the ablation shape. In contrast, higher flow rates of more than 10 ml/min were required to detect recognizable cooling effects centrally when the vessel was in close proximity to the ablation centre. As local energy density is highest around the antenna, higher flow rates were necessary to induce a visible central heat sink effect. At a maximal flow rate of 500 ml/min, 19.2% of the introduced energy was absorbed through the vessel. However, the flow velocity seems to play a secondary role in the formation of vascular cooling effects in relation to the antenna-vessel spacing. Although, centrally located vessels within an ablation only cause minor cooling effects in the ablation centre, these vessels cross the ablation border. In these cases, distinct cooling effects occur at both ablation entry points. This resulted in more pronounced absolute cooling volumes in relation to the experimental setups with only peripherally located vessels. If cooling effects occur centrally, there is a risk of an alternation of ablation shape and volume. In doubtful cases, temporary interruption of the liver perfusion through a laparoscopic or open surgical Pringle manoeuvre must be considered^16^.

Our study has several limitations. Direct clinical applicability of the presented results is difficult with an ex vivo model. Although HCC tumour models exist for porcine liver, we performed the experiments on native porcine liver for ethical and cost-effective reasons^23,24^. However, the electrical and thermal properties of tumour tissue have been examined. As a result the differences to native liver tissue can be calculated mathematically and subsequently be transferred to a tumour model^25^. Another limitation is the use of a single glass tube to simulate a liver vessel^12^. For this reason, no statement can be made about the diffuse cooling effect or the combination of both the diffuse and directional cooling effect. Even though an evaluation of the interaction between the diffuse and directional cooling effect would be desirable, accurate and simultaneous evaluation of vessel diameters, flow rates and three-dimensional vasculature in the liver are necessary for such an analysis. Due to this complexity, these interactions can hardly be solved experimentally. In comparison to the complex anatomical vessel structure of the liver, the application of only one vessel ensured a standardized experimental set up. Moreover, glass has a similar heat conductivity to natural liver tissue and does not interfere with heat distribution^26^. However, a decrease in ablation volume can be seen in the experiments without cooling volume (0 ml/min) with increasing A–V distance. This could indicate that the glass tube does cause a small cooling effect, which is more pronounced at the edge of the ablation than in the centre and thus leads to a decrease in ablation volume. For our study, this effect is not relevant since the cooling effects were always examined among identical A–V distances. In the present study only one vessel diameter was investigated, so that no statement can be made about the effect of vessel diameter in three-dimensional space. However, previous studies have shown that the vessel diameter does not influence the cooling effect^12,27^. A single energy input (100 W, 5 min) was analysed in this study. It can be assumed that cooling effects would vary for different energy inputs and hence affect the resulting ablation volumes. For this reason, the cooling effects should not be seen in absolute terms as a function of the A–V distance, but in relation to the respective expected ablation size and thus the position of the vessel relative to the ablation edge. All experiments were carried out at room temperature. Generally, more pronounced cooling effects seem likely at room temperature in comparison to body temperature due to a higher thermal gradient. However, we could show in previous experiments for RFA that comparable results to those at body temperature^11^.

MWA has become an established minimally invasive procedure in recent years. However, complete verification or exact prediction of cooling effects is still difficult in MWA in vivo. For this reason, experimental studies such as this one are necessary to understand the cooling effects in MWA and provide data to optimize existing numerical simulations to support clinicians in therapy planning. Exact knowledge of the impact of vascular cooling is essential to make MWA a safer and more reliable therapy option.

Vascular cooling effects have to be taken into account in hepatic MWA. The cooling effect is mostly dependent on the distance between the vessel to the ablation centre. Distinct cooling effects occur at the ablation border even at low flow rates and distinctly influence ablation shape. Central cooling effects are less likely but may arise at higher flow rates and result in smaller ablation volumes. Therefore, a surgical Pringle manoeuvre should be considered when complete ablation does not seem probable.

## Materials and methods

### Experimental setup

Porcine livers were obtained from an abattoir directly after slaughter. In order to prevent desiccation and cooling, livers were stored in airtight and thermally insulated containers. All experiments were carried out at room temperature within 6 h post mortem. The livers were cut into pieces of approximately 5 × 5 × 7 cm, suitable for a subsequent ablation.

Microwave ablations were performed with the Emprint™ MWA system (Covidien, Boulder, CO, USA), which is regularly used in clinical practice^28^. The system consists of a MWA-generator which uses a frequency of 2.45 GHz with a maximum power of 100 W. The generator is connected to a compatible internally cooled fibre glass microwave antenna through a flexible coaxial cable. The applied antenna had a diameter of 2.0 mm and a total length of 20 cm including an active length of 25 mm (Emprint, Covidien, Boulder, CO, USA). Internal cooling of the antenna was induced with a roller pump attached to the generator using saline solution (Fresenius NaCl 0.9%, 1000 ml, Plastipur^®^, Fresenius Kabi Deutschland GmbH, Bad Homburg, Deutschland) at a continuous flow rate of 60 ml/min. Total ablation power was set to 100 W for 5 min according to the manufacturers recommendation to obtain an ablation size of 3.6 × 3.2 cm (length × width). According to literature the presence of vessels with a diameter of at least 3 mm in size adjacent to hepatic tumours is a strong predictor of incomplete tumour destruction^21^. Hence, a natural liver vessel was simulated with a glass tube (hereafter referred to as “vessel”) with an outer diameter of 5.0 mm and an inner diameter of 3.0 mm^11,13,29,30^. However, we could demonstrate in a previous 2D-study that a vessel diameter > 3 mm has no influence on the cooling effect in MWA^27^. As the use of glass tubes with a diameter < 3 mm is not possible for technical reasons, only one vessel diameter (3 mm) was investigated. The glass tube (V) was inserted into the liver tissue at distances of 2.5, 5.0 and 10.0 mm (A–V distance), parallel to the microwave antenna (A). A peristaltic pump ensured a constant perfusion of the vessel (flow rates ≤ 5 ml/min: Minipuls^®^ 3, Abimed, GILSON, USA; flow rates ≥ 10 ml/min: Watson-Marlow™ 323E/D, Bredel Pumps, Falmouth, Cornwall, England). Seven different flow volumes were evaluated (each n = 6): 0, 1, 2, 5, 10, 100 und 500H_2_O ml/min. Therefore, 126 ablations were planned. The temperature difference (∆T) of the cooling liquid was measured by taking the temperature before (room temperature) and after MWA in the test series with perfusion rates of 100 and 500 ml/min to determine the energy loss (∆Q) through the vessel (∆Q = m × c_aqua_ × ∆T; m = mass of the cooling volume; c_aqua_ $\frac{kJ}{kg\times K}$).

A custom-made aiming device (acrylic glass) ensured an accurate positioning of the microwave antenna and the vessel into the liver sample (Fig. 4a). The device enabled a standardized division exactly through the ablation centre, orthogonally to the antenna. All ablations were embedded in Tissue Tek^®^ O.C.T.™ (Sakura Finetek Germany GmbH, Staufen, Germany) and immediately frozen in liquid nitrogen before being stored at − 80 °C. A cryostat (CryoStar™ NX70 Cryostat, ThermoFisher Scientific, Waltham, USA) was used to trim each half of the ablation in 50 µm steps at − 12 °C. A photograph (D5100 model, Nikon Corporation, Tokio, Japan) was taken every 2.0 mm next to a millimetre scale for a subsequent 3D-reconstruction.

### 3D-analysis

**Figure 5:**
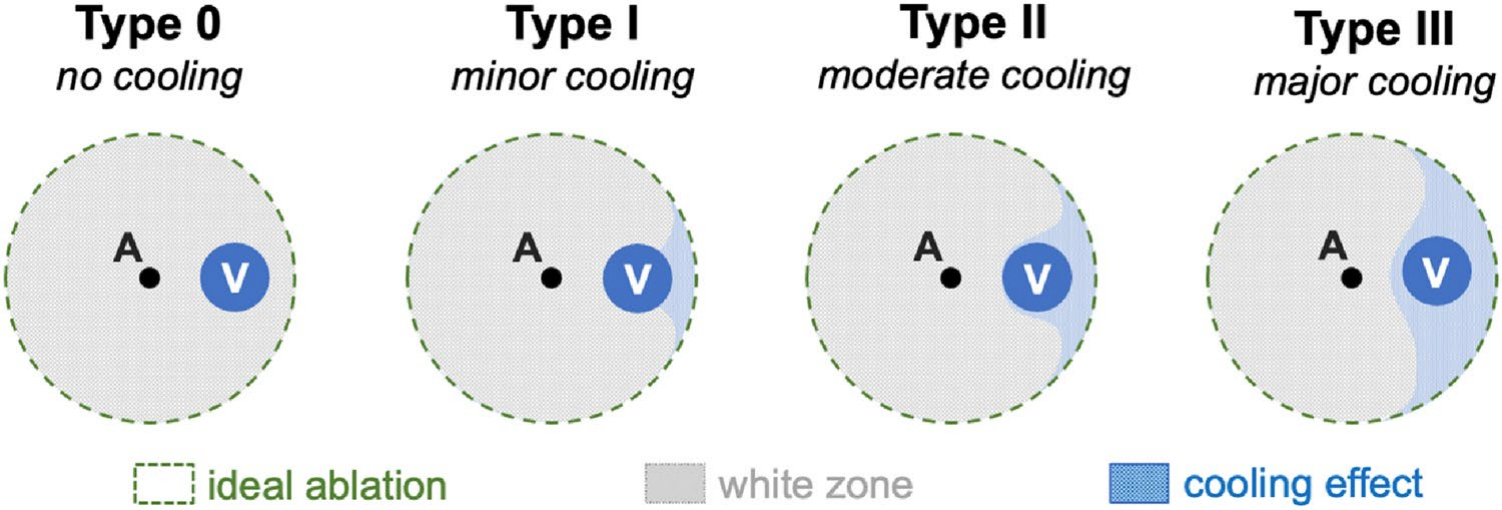
The effect of vascular cooling on ablation shape was assessed using a semiquantitative classification scheme: Type 0: no cooling, Type I: minor cooling, Type II: moderate cooling, Type III: major cooling.

Each ablation half was sectioned into a maximum of ten slices beginning from the ablation centre (Fig. 4b). Ablation shapes of each slice were assessed qualitatively by comparing the ablation shape to a round and homogenous ablation for all flow rates at different vessel to antenna distances. In addition, each ablation slice was semi-quantitatively classified into one of four different ablation types depending on the observed cooling effect as described previously by Frericks et al.^31^ (Fig. 5):

- No cooling: the ablation shape is not affected by the vessel
- Minor cooling: the ablation shape is slightly indented in vessel vicinity
- Moderate cooling: the ablation shape is strongly indented in vessel vicinity, but the vessel is still partly enclosed by the ablation
- Major cooling: the ablation is strongly indented in vessel vicinity, but the vessel is outside of the ablation

For a quantitative analysis, ablation images were transferred to a custom-made software tool (MWANecrosisMeasurement, Fraunhofer Institute for Digital Medicine MEVIS, Bremen, Germany). The millimetre paper photographed beside the ablation cross sections was used to calibrate the images. Afterwards, the antenna and vessel as well as the central ablation zone, known as the “white zone” (WZ), were marked manually. The WZ represents the area of the ablation which corresponds to irreversibly damaged tissue^14,32^. In clinical practice the tumour should be encircled by this zone to ensure therapeutic success. The WZ is macroscopically identified by its pale grey colour. The so-called adjoining “red zone” (RZ) is defined by a combination of ablated as well as viable tissue and merges into native liver tissue. Since vital cells are preserved in the red zone, tumour recurrence cannot be ruled out in this area. The software computed the maximum radius and area of the respective ablation cross sections after the WZ and RZ were manually outlined. Based on these measured values the ablation and cooling volumes were calculated. The volume of each ablation (V_x_) was reconstructed by applying the formula of a truncated cone (V_x_ = $\frac{{A}_{1}+ {A}_{2}}{2}\times {d}_{2mm}$+ ⋯ + $\frac{{A}_{n-1}+ {A}_{n}}{2} \times {d}_{2mm}$). This formula contains the determined ablation areas (A_1−n_) of each slice as well as the spacing between the sectional 2 mm planes (d_Δ2mm_). In order to determine the cooling volume, an “idealized ablation” with no cooling effects was created. This ideal ablation was generated by using the maximum ablation radius (r_max_) of the respective ablation slice. The cooling area is defined by the difference between the idealized ablation and the actual ablation area. As cooling effects are expected in close proximity to the vessel, only the ablation half containing the vessel was included in the analysis to ensure that cooling effects could be observed as isolated as possible around the cooling vessel (Fig. 4c). Subsequently, the cooling volume was calculated using the truncated cone formula stated above.

### Statistical analysis

Statistical analysis was performed with SPSS (IBM SPSS Statistics, version 27 for Windows, Armonk, USA). Results are presented as median (minimum–maximum). The Mann–Whitney U test was used for a comparison between two independent groups. The Kruskal–Wallis test was applied for a comparison between more than two independent groups. A Bonferroni correction was carried out to increase accuracy for the simultaneous application of multiple testing. Therefore, the level of significance was set as p ≤ 0.008 (two sided). p-values between 0.008 and 0.05 were interpreted as a trend (not significant).

## Data Availability

The datasets analysed in our study are available from the corresponding author on reasonable request.
